# Hepatotoxicity of the Major Anthraquinones Derived From Polygoni Multiflori Radix Based on Bile Acid Homeostasis

**DOI:** 10.3389/fphar.2022.878817

**Published:** 2022-05-18

**Authors:** Li Kang, Dan Li, Xin Jiang, Yao Zhang, Minhong Pan, Yixin Hu, Luqin Si, Yongjun Zhang, Jiangeng Huang

**Affiliations:** ^1^ School of Pharmaceutical Science, South-Central MinZu University, Wuhan, China; ^2^ School of Pharmacy, Tongji Medical College, Huazhong University of Science and Technology, Wuhan, China; ^3^ National Demonstration Center for Experimental Ethnopharmacology Education, South-Central MinZu University, Wuhan, China; ^4^ Department of Pharmacy, Shenzhen University General Hospital, Shenzhen, China; ^5^ College of Pharmacy, Key Laboratory of Xinjiang Phytomedicine Resource and Utilization, Ministry of Education, Shihezi University, Shihezi, China; ^6^ The Third Affiliated Hospital of School of Medicine, Shihezi University, Shihezi, China

**Keywords:** Polygoni Multiflori Radix, anthraquinones, bile acids, homeostasis, transporter

## Abstract

Polygoni Multiflori Radix (PMR), the dried root of *Polygonum Multiflorum* Thunb., has been widely used as traditional Chinese medicines in clinical practice for centuries. However, the frequently reported hepatotoxic adverse effects hindered its safe use in clinical practice. This study aims to explore the hepatotoxic effect of PMR extract and the major PMR derived anthraquinones including emodin, chrysophanol, and physcion in mice and the underlying mechanisms based on bile acid homeostasis. After consecutively treating the ICR mice with PMR extract or individual anthraquinones for 14 or 28 days, the liver function was evaluated by measuring serum enzymes levels and liver histological examination. The compositions of bile acids (BAs) in the bile, liver, and plasma were measured by LC-MS/MS, followed by Principal Component Analysis (PCA) and Partial Least Squares Discriminate Analysis (PLS-DA). Additionally, gene and protein expressions of BA efflux transporters, bile salt export pump (Bsep) and multidrug resistance-associated protein 2 (Mrp2), were examined to investigate the underlying mechanisms. After 14-day administration, mild inflammatory cell infiltration in the liver was observed in the physcion- and PMR-treated groups, while it was found in all the treated groups after 28-day treatment. Physcion and PMR extract induced hepatic BA accumulation after 14-day treatment, but such accumulation was attenuated after 28-day treatment. Based on the PLS-DA results, physcion- and PMR-treated groups were partially overlapping and both groups showed a clear separation with the control group in the mouse liver. The expression of Bsep and Mrp2 in the physcion- and PMR-treated mouse liver was decreased after 14-day treatment, while the downregulation was abrogated after 28-day treatment. Our study, for the first time, demonstrated that both PMR extract and tested anthraquinones could alter the disposition of either the total or individual BAs in the mouse bile, liver, and plasma *via* regulating the BA efflux transporters and induce liver injury, which provide a theoretical basis for the quality control and safe use of PMR in practice.

## 1 Introduction

Polygoni Multiflori Radix (PMR), originated from the tuberous root of *Polygonum multiflorum* Thunb., is a well-known traditional Chinese medicine and has been commonly used in clinical practice in either raw or processed forms for different indications such as detoxification, carbuncle elimination, malaria prevention, bowel relaxation, hair-darkening, nourishment of the liver and kidney, and so on (Chinese Pharmacopoeia Commission, 2015). Although PMR was widely used in clinical practice or as a tonic, a series of cases of hepatic adverse effects associated with PMR or the herbal medicine products containing PMR were constantly reported (But et al., 1996; Park et al., 2001; Cárdenas et al., 2006; Laird et al., 2008; Cho et al., 2009; Jung et al., 2011; Dong et al., 2014). Accordingly, the supervisions of clinical usage of PMR have been conducted by the drug regulatory agencies in Canada, Australia, the United Kingdom, and China (Canadian Adverse Reaction Newsletter, 2003; Medicines and Healthcare products Regulatory Agency, 2006; Complementary Medicines Evaluation Committee, 2008; China Food and Drug Administration, 2014). Therefore, the safe and effective use of PMR in clinical practice has become an important and urgent problem which needed to be resolved.

According to previous reports, more than 100 components from PMR had been isolated and identified including stilbenes, anthraquinones, dianthrones, flavonoids, and phenolic acids (Yang et al., 2016; Wang et al., 2017; Yang et al., 2017; Yang et al., 2018b; Yang et al., 2019). After oral administration in rats with PMR extract, 41 compounds were detected in the rat plasma with stilbenes and quinones as the major components (Wang et al., 2017). The stilbenes were reported to possess anti-oxidative, anti-aging, anti-tumor, and hepatoprotective activities (Lin et al., 2015; Wu et al., 2017; Zhang and Chen, 2018), while the anthraquinones were mainly used for anti-bacterial, anti-fungal, and anti-cancer activities (Lin et al., 2015; Dong et al., 2016; Xun et al., 2019). As for the aspect of toxicity, several anthraquinones including emodin and emodin-8-*O*-*β*-D-glucoside, dianthrones such as (*C*is)-emodin-emodin dianthrones and (*T*rans)-emodin-emodin dianthrones as well as stilbenes such as (Trans)-2, 3, 5, 4′-tetrahydroxy stilbene-2-*O*-*β*-d-glucopyranoside and (*C*is)-2, 3, 5, 4′-tetrahy droxystilbene-2-*O*-*β*-d-glucopyranoside have been identified as the potential components for the hepatotoxicity of PMR (Yang et al., 2021). Moreover, among the known anthraquinones, emodin, physcion, and chrysophanol were the most detectable components in the plasma after oral consumption of PMR extract (Li et al., 2020a). These anthraquinones were also reported to induce liver toxicity *via* multiple mechanisms including reactive oxygen species induced endoplasmic reticulum stress and mitochondrial dysfunction as well as cytochrome c release mediated apoptosis (Lai et al., 2009; Qu et al., 2013). In addition, Zhang et al. (2020a) have also suggested that the stilbene in PMR could synergize the idiosyncratic hepatotoxicity of anthraquinones from PMR.

Bile acids (BAs), the major components of bile, are formed from cholesterol through various enzymatic reactions in the hepatocytes (García-Cañaveras et al., 2012). The homeostasis of BAs plays a crucial role in the metabolism of lipid, glucose, and energy (Verkade et al., 1995; Houten et al., 2006; Kuipers et al., 2014), and the disorder of BAs would cause hepatobiliary diseases (Stanimirov et al., 2015), gallstones (Berr et al., 1992), or gastrointestinal cancers (Bernstein et al., 2005). In recent years, several metabolomic (Dong et al., 2015; Zhang et al., 2015; Xia et al., 2017; Yan et al., 2020; Zhang et al., 2020b) and transcriptome (Jiang et al., 2018) analyses demonstrated that the toxicity mechanisms of PMR-induced liver injury may relate to the disruption of BA homeostasis and the metabolism of energy, amino acids, fatty acids, and lipids. Moreover, cholestasis was also observed after treating the rats with PMR extract for 28 days (Wang et al., 2015). In addition, our group has demonstrated that emodin and physcion, the major components of PMR, could alter BA disposition and induce BA accumulation in the sandwich-cultured rat hepatocytes (Kang et al., 2017). However, whether these anthraquinones have any influence on the BA homeostasis contributing to the PMR-induced liver injury after its long-term exposure *in-vivo* remains unknown. In this study, to investigate the hepatotoxicity of PMR extract and three individual anthraquinone from PMR, namely, emodin, chrysophanol, and physcion, the ICR mice were consecutively administered with PMR extract or the pure anthraquinones for 14 or 28 days.

## 2 Materials and Methods

### 2.1 Chemicals and Reagents

Emodin, chrysophanol, physcion, and 2,3,4’,5-tetrahydroxystilbene-2-*O*-*β*-D-glucoside (TSG) (purities >98%) were obtained from Nanjing Jingzhu Co. Ltd. (Jiangsu, China). The aspartate aminotransferase (AST), serum alanine aminotransferase (ALT), triglyceride (TG), and γ-glutamine transferase (γ-GGT) assay kits were supplied by Nanjing Jiancheng Bioengineering Institute (Jiangsu, China). The bicinchoninic acid (BCA) protein assay kit was purchased from Beyotime (Jiangsu, China). Individual BA together with deuterium-labelled glycol-chenodeoxycholic acid (d4-GCDCA) were obtained as previously described (Huang et al., 2011). All other chemicals and reagents were of analytical grade and readily available from commercial sources.

### 2.2 Preparation of the Ethanol Extract of PMR

The raw PMR from Dabashan, Sichuan Province, identified by Prof. Jianping Wang, Huazhong University of Science and Technology, was used in our study. The PMR extract was prepared by crushing the raw materials into powder, followed by refluxing for 48 h with 75% (v/v) ethanol at a solid–liquid ratio of 1:6 and filtration. The extraction was repeated five times and the combined filtrates were concentrated to remove the ethanol and then lyophilized for further use.

### 2.3 Identification and Determination of Major Anthraquinones From PMR Ethanol Extract

The major components from PMR extract were identified based on the previous study (Li et al., 2021). Briefly, 0.2 g PMR extract was sonicated with 25 ml acetone for 1 h and centrifuged at 1800 g for 10 min. The supernatant was evaporated to dryness, reconstituted with 2 ml methanol (MeOH), and filtered for HPLC analyses. Additionally, the contents of major PMR derived anthraquinones including emodin, chrysophanol, and physcion in the solution were determined *via* HPLC and the contents of all the analytes were calculated as percentage (%, g/100 g crude material).

### 2.4 Animals and Treatment

Male ICR mice, weighing 30 ± 5 g, were obtained from the Laboratory Animal Center of Tongji Medical College, Huazhong University of Science and Technology (HUST, Wuhan, China). The mice were acclimatized to the environmentally controlled condition (temperature of 22–27°C, relative humidity of 40%–70%, and 12 h light/dark cycle) for 7 days with free access to food and water before and during the treatment interval. The anthraquinones and PMR extract powder was dissolved in 0.5% (w/v) CMC-Na each day before treatment, and the guideline for the volume of oral administration is 1 ml/100 g body weight in mice. Eighty ICR mice were randomly divided into 10 groups (n = 8): five of the groups were consecutively orally administered with 0.5% (w/v) carboxymethylcellulose sodium (CMC-Na) solution, 100 mg/kg emodin, 100 mg/kg chrysophanol, 100 mg/kg physcion, or 1.5 g/kg PMR extract (equivalent to 30 g crude material/kg) for 14 days; the other five groups received the same doses for 28 days. Twenty-four hours after the last dose, the animals were sacrificed and the blood, bile, and liver tissues were collected for further analyses. All animal experiment procedures were approved by the Institutional Animal Care and Use Committee of Tongji Medical College, HUST.

### 2.5 Biochemical Analysis and Histopathological Examination

The blood samples were collected and centrifuged at 1,000 g for 10 min, and the supernatant sera were obtained. Biomarkers, including AST, ALT, TG, and γ-GGT in the serum, were determined using the commercially available kits according to the manufacturer’s instructions. Moreover, the livers fixed in 4% formalin were embedded in paraffin and sliced to a thickness of 5 µM. After staining with hematoxylin and eosin (H&E), the slices were examined under a light microscope for structural changes.

### 2.6 Sample Preparation

The sample preparation procedure was based on our previous method for the analysis of BAs with slight modification (Huang et al., 2011). For the plasma samples, the d4-GCDCA (internal standard, IS) solution (10 μl, 1 μg/ml) was spiked into 100 μl plasma, followed by the addition of 1 ml ice-cold alkaline acetonitrile (ACN) containing 5% (v/v) NH_4_OH, vortexed for 10 min and centrifuged at 16,000 × g for 10 min. The supernatant was aspirated, evaporated, and reconstituted in 100 μl of 50% MeOH in water. For the liver samples, after homogenizing approximately 100 mg of liver with 2 volumes of H_2_O, 10 μl IS solution was spiked into 300 μl liver homogenate and 2 ml of ice-cold 5% NH_4_OH in ACN was added. After vortexing for 10 min, the samples were centrifuged at 16,000 × g for 10 min. The supernatant was aspirated, and the pellet was extracted with another 1 ml of ice-cold 5% NH_4_OH in ACN. The supernatants were pooled, evaporated to dryness, and reconstituted with 100 μl 50% MeOH in water. As for the bile samples, the Supelclean™ LC-18 SPE cartridges (Sigma–Aldrich, St. Louis, MO) were used for sample extraction. First, the bile samples were diluted 20- and 2000-fold with deionized water. Then, 100 μl of diluted bile samples spiked with 10 μl IS was loaded onto SPE cartridges which were preconditioned with 2 ml MeOH, followed by 2 ml H_2_O. Thereafter, the loaded cartridges were washed with 2 ml H_2_O and eluted with 4 ml MeOH. Finally, the eluted solution was evaporated to dryness and reconstituted with 100 μl 50% MeOH in water.

### 2.7 LC-MS/MS Analysis

Simultaneous determination of individual endogenous BAs in the bile, liver homogenate, and plasma was performed according to our previously published liquid chromatography with the tandem mass spectrometry (LC-MS/MS) method with slight modifications (Huang et al., 2011). Chromatographic separation was achieved on a Waters ACQUITY HSS T3 column (2.1 × 100 mm, 1.8 μm; Waters, United States). The mobile phase consisted of 7.5 mM ammonium acetate, adjusted to pH 7.0 using 10 M ammonium hydroxide (mobile phase A) and MeOH (mobile phase B) and the flow rate was 0.3 ml/min. The elution gradient profile started from 50% mobile phase B, linearly increased to 90% mobile phase B in 20 min, held at 90% for 2 min, and brought back to 50% in 0.01 min followed by 4 min re-equilibration. The BA analysis was performed on a Shimadzu Prominence UFLC system (Shimadzu Corporation, Japan) and an API 4000 QTrap^®^ triple quadrupole mass spectrometer (AB SCIEX, United States) with an electrospray ionization (ESI) source. All the BAs were measured in negative ionization mode using mass transitions listed in our previous report. The main working parameters for the MS were set as: capillary voltage, −4000 V; source temperature, 600°C; curtain gas flow, 20 psi; nebulizer gas flow, 35 psi; and collision energy, high.

### 2.8 Quantitative Real-Time PCR

Total RNA was extracted from liver using TRIzol reagent according to the manufacture’s instruction (Invitrogen, United States). The RNA samples were reversed-transcribed into complementary DNA (cDNA) *via* Fermentas RevertAid First Strand cDNA Synthesis Kit (ThermoFisher, United States) according to the manufacturer’s protocol. The cDNA was amplified using an ABI Step-One Sequence Detection System (Applied Biosystems, United States) with SYBR Green PCR Master Mix (Bio-Rad, United States). The gene expression was determined by normalization with control gene Gapdh using the ΔΔCt method and the primer sequences are set as: Bsep (Gene ID: 27413, forward: 5′-GCT​GCC​AAG​GAT​GCT​AAT​GC-3′ and reverse: 5′-TTG​GGT​TTC​CGT​ATG​AGG​GC-3′), Mrp2 (Gene ID: 12780, forward: 5′- CCT​TGG​GCT​TTC​TTT​GGC​TC-3′ and reverse: 5′-ACA​CAA​CGA​ACA​CCT​GCT​TG-3′), and Gapdh (Gene ID: 14433, forward: 5′-GTC​GGT​GTG​AAC​GGA​TTT​GG-3′ and reverse: 5′-TCA​GAT​GCC​TGC​TTC​CCA​TTC-3′).

### 2.9 Western Blotting

Total protein in the liver was extracted with RIPA Lysis Buffer (Beyotime) containing 1 mM PMSF, and the total protein concentration was determined using the BCA protein assay kit. An equal amount of proteins (50 μg) was separated by electrophoresis on sodium dodecyl sulfate/8.75% polyacrylamide gel electrophoresis and transferred to nitrocellulose blotting membranes (Millipore, United States) for 2 h through wet transfer method using Mini Trans-Blot (Bio-Rad, United States). Subsequently, the membranes were blocked with 5% nonfat milk dissolving in 0.5% TBST buffer for 1.5 h and then incubated overnight at 4°C with specific primary antibodies including Bsep (goat, Santa Cruz Biotech), Mrp2 (rabbit, Abcam), and β-actin (mouse, Proteintech). After washing the membrane with 0.5% TBST buffer for three times, the membranes were further incubated with corresponding secondary antibodies at room temperature for 1 h. The protein bands were visualized using an enhanced chemiluminescence detection system (Millipore, United States).

### 2.10 Data Processing and Statistical Analysis

All values were indicated as mean ± standard deviation (mean ± SD). Statistical analyses were carried out using GraphPad Prism 7.0 (GraphPad Software Inc., United States). Differences between the two groups were analyzed by Student’s t-test, while multiple comparisons were carried out by one-way ANOVA followed by Dunnett’s post hoc test. Differences with a probability value (P) < 0.05 were statistically significant. MetaboAnalyst 5.0 was used to perform the Principal Component Analysis (PCA) and Partial Least Squares Discriminant Analysis (PLS-DA) for the evaluation of the differences of the BAs in the liver, bile, and plasma between the control and treatment groups.

## 3 Results

### 3.1 HPLC Chromatogram of PMR Extract and the Contents of Major Anthraquinones

The HPLC chromatogram of the PMR extract is shown in Supplementary Figure S1. The retention time of TSG, one of the major quality control marker components of PMR, was 4.131 min and that of emodin, chrysophanol, and physcion was 9.154, 10.157, and 11.886 min, respectively. The contents of emodin, chrysophanol, and physcion were 0.047 ± 0.00026, 0.0063 ± 0.00046, and 0.014 ± 0.00025 (%, g/100 g crude material), respectively.

### 3.2 Biochemical and Histopathological Analysis

**FIGURE 1:**
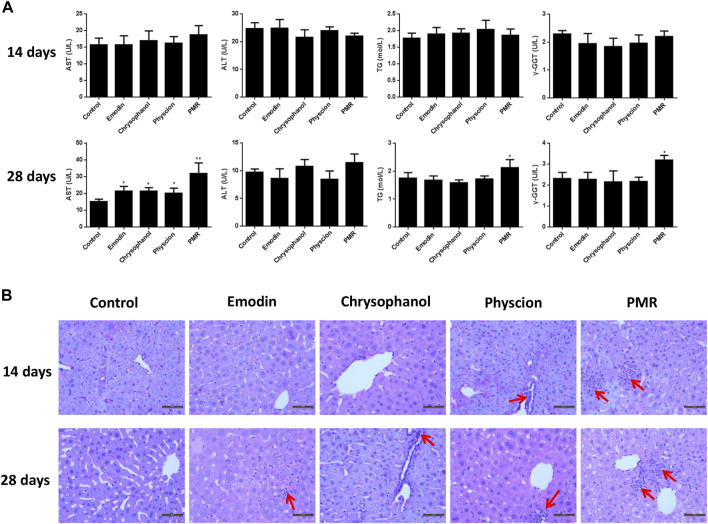
Serum levels of AST, ALT, TG, and γ-GGT **(A)** and typical liver histopathological section photos (**(B)** Hematoxylin and eosin stained, original magnification ×400) in different groups of mice following 14- or 28-day treatment with anthraquinones or PMR extract. Inflammatory cell infiltration was indicated by the solid arrow. Data were presented as mean ± SD (n = 8). *p < 0.05, **p < 0.01 compared with control group.

To assess the hepatotoxicity of the PMR extract and its major components, several biochemical biomarkers indicating liver injury in serum were investigated. As shown in Figure 1A, after administration for 14 days, ALT, AST, TG, and γ-GGT levels showed no significant change in all the groups. After treating the animals for 28 days, it was noted that the AST levels were significantly increased in all the treated groups. Additionally, the levels of TG and γ-GGT were also increased in the PMR-treated group. The histopathological analysis of the liver (Figure 1B) indicated mild inflammatory cell infiltration after treating the mice with physcion or PMR extract for 14 days. However, there was no lesion observed in emodin- or chrysophanol-treated groups after 14-day treatment. With the treatment time prolonging to 28 days, inflammatory cell infiltration was observed in all the treated groups as shown in Figure 1B.

### 3.3 Quantitative Analysis of BAs

#### 3.3.1 Alteration of Individual BA in the Mouse Liver, Bile, and Plasma

**FIGURE 2:**
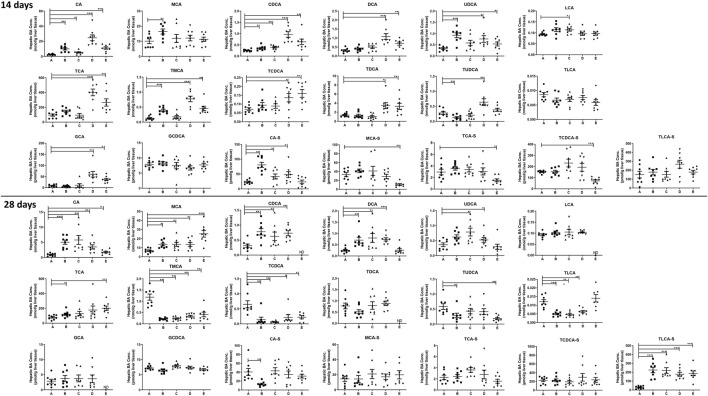
Concentration of the individual BAs in the mouse liver after the anthraquinones or PMR consumption for 14 or 28 days (A: control, B: emodin, C: chrysophanol, D: physcion, E: PMR). The data are expressed as the mean ± SD (n = 6–8). *p < 0.05, **p < 0.01, ***p < 0.001 compared with control group, ND: not detected.

To investigate the changes of BA profiles after treating the mice with PMR extract or its major anthraquinones, the abundance of 36 BAs in the plasma, liver homogenate, and bile were determined. After treating the mice for 14 or 28 days, 19 different BAs were detected in the liver homogenate. As shown in Figure 2, compared with the control group, the concentrations of cholic acid (CA), chenodeoxycholic acid (CDCA), deoxycholicacid (DCA), ursodeoxycholic acid (UDCA), tauro-cholic acid (TCA), tauro-β-muricholic acid (TMCA), tauro-chenodeoxycholic acid (TCDCA), tauro-deoxycholic acid (TDCA), and glyco-cholic acid (GCA) in the liver were significantly increased after treating the mice with physcion and PMR for 14 days. In addition, it was found that the concentrations of sulfated BAs including β-muricholic acid-sulfate (MCA-S), tauro-cholic acid-sulfate (TCA-S), and tauro-chenodeoxycholic acid-sulfate (TCDCA-S) were significantly decreased after treating the mice with PMR for 14 days. In the emodin-treated group, the significant accumulation of CA, β-muricholic acid (MCA), CDCA, UDCA, TMCA, and cholic acid-sulfate (CA-S) were observed after treating the mice for 14 days. As for chrysophanol, only the concentrations of CA, CDCA, lithocholic acid (LCA), and CA-S were increased. After consecutively treating the mice for 28 days, the content of CA, MCA, and tauro-lithocholic acid-sulfate (TLCA-S) were significantly increased in all the treated groups. In the meantime, the quantities of TMCA and TCDCA in the liver were significantly decreased in all the treated groups. After treating the mice with these anthraquinones for 28 days, hepatic accumulation of CDCA and DCA was increased, while tauro-lithocholic acid (TLCA) in the liver were significantly decreased. In addition, the quantities of TCA were increased, whereas tauro-ursodeoxycholic acid (TUDCA) was decreased in the emodin- or PMR-treated groups. Chrysophanol and physcion were found to increase the UDCA levels in the mouse liver.

**FIGURE 3:**
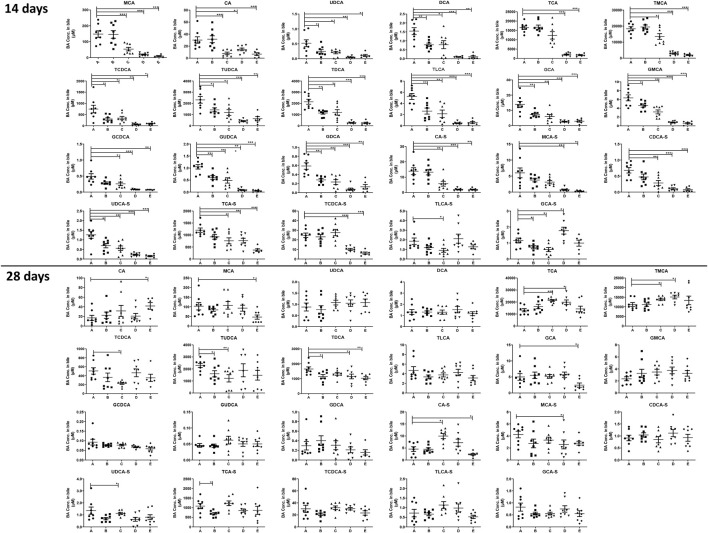
Concentration of the individual BAs in the mouse bile after the anthraquinones or PMR consumption for 14 or 28 days (A: control, B: emodin, C: chrysophanol, D: physcion, E: PMR). The data are expressed as the mean ± SD (n = 6–8). *p < 0.05, **p < 0.01, ***p < 0.001 compared with control group.

After treating the mice for 14 days, it was noted that almost all the detected BAs in the bile in our current study were significantly decreased after treating the mice with anthraquinones or PMR extract (Figure 3). However, after treating the mice for 28 days, the inhibition of active BA excretion was significantly attenuated and the bile level of a fewer number of BAs was found to be decreased compared to the 14-day treatment groups. Only the decrease of TUDCA, TDCA, and TCA-S in the emodin-treated group; TCDCA, TUDCA, and ursodeoxycholic acid-sulfate (UDCA-S) in the chrysophanol-treated group; TDCA and MCA-S in the physcion-treated group; as well as MCA, TDCA, GCA, and CA-S caused by PMR were observed. Moreover, the content of TCA, TMCA, and CA-S in the chrysophanol group; TCA and TMCA in the physcion group; and CA in the PMR group were significantly increased.

As for the plasma (Supplementary Figure S2), only seven BAs were detected and it was found that except for β-muricholic acid (MCA), emodin had no significant influence on the detectable BAs after treating for 14 days. However, another two anthraquinones, chrysophanol and physcion, were found to decrease the content of CDCA, TMCA, and TLCA-S. In the PMR-treated group, the quantity of TCA was increased and the concentrations of MCA, TMCA, TDCA, and TLCA-S were decreased after treating for 14 days. After treating the mice for 28 days, the concentrations of CA were decreased in the chrysophanol and physcion treatment groups. Moreover, physcion was found to significantly elevate the concentrations of TDCA in the plasma after 28-day administration.

#### 3.3.2 Disruption of the Total BAs in Mouse Liver, Bile, and Plasma

**FIGURE 4:**
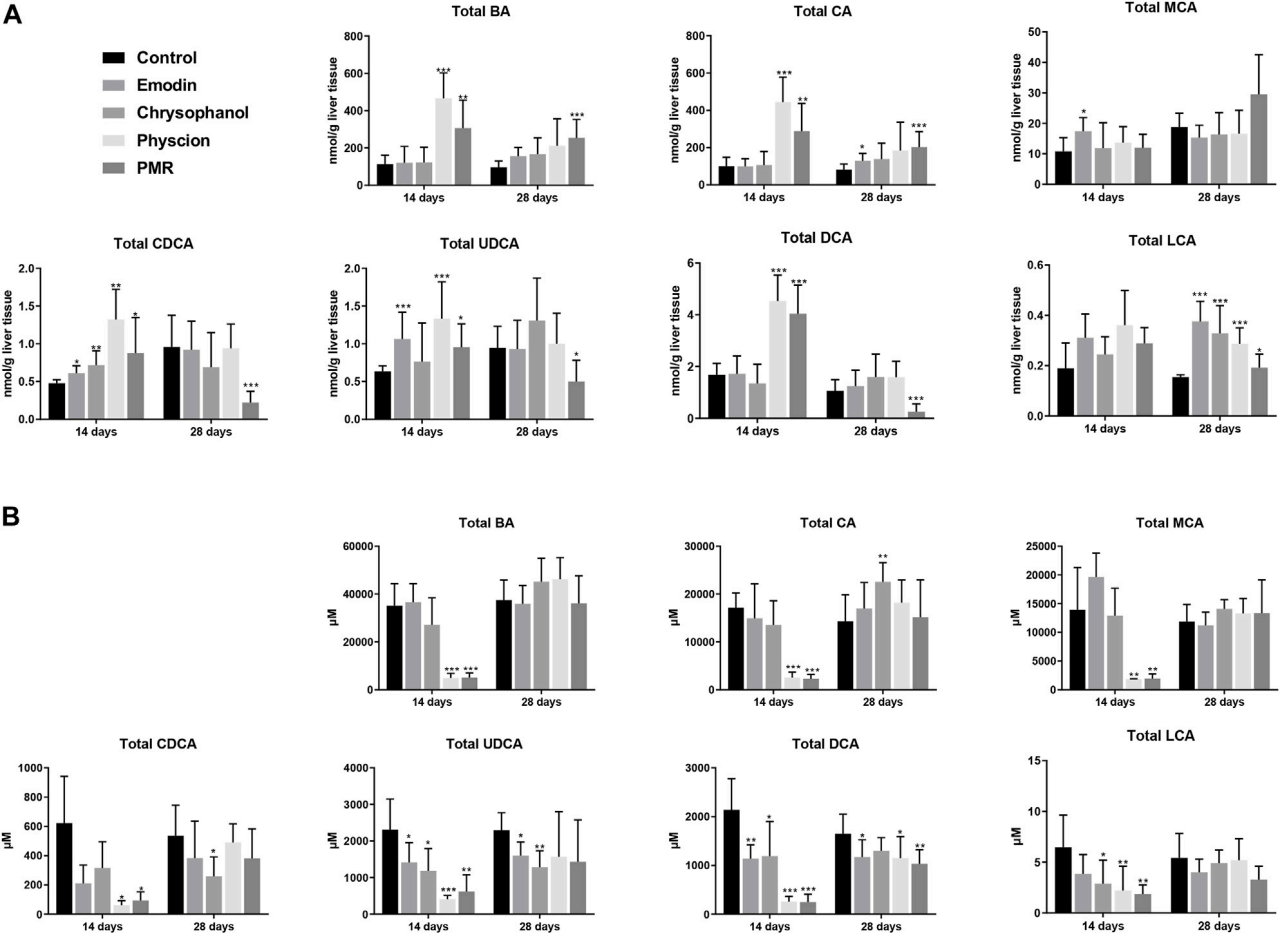
Comparison of the total BA, total CA, total MCA, total CDCA, total UDCA, total DCA, and total LCA in the mouse liver **(A)** and bile **(B)** between different groups of mice following 14- or 28-day treatment with anthraquinones or PMR extract. The data are expressed as the mean ± SD (n = 6–8). *p < 0.05, **p < 0.01, ***p < 0.001 compared with control group.

As shown in Figure 4A, compared with the control group, it was noted that the total BAs in the liver was significantly increased after treating with physcion or PMR extract for 14 days. In the meantime, the total BAs in the bile were significantly decreased in these two groups (Figure 4B). Moreover, the content of the total BAs in the plasma was also significantly decreased after treating with chrysophanol or physcion for 14 days (Supplementary Figure S3). However, the accumulation of total BAs in the liver after treating with physcion for 28 days was attenuated in the absence of significant difference with the control group (Figure 4), while the total BAs in the liver after treatment with PMR extract for 28 days were significantly increased in comparison with the control group (Figure 4). In addition, the total BAs in the bile after treating with anthraquinones or PMR extract for 28 days showed no difference with the control group. Similar to the total BAs, the total CA, total CDCA, total UDCA, and total DCA in the liver were significantly increased in the physcion- and PMR-treated groups after 14-day dosing (Figure 4A), while the concentrations of these total BAs in the bile were decreased in these two groups (Figure 4B). Moreover, in the liver, the total CDCA in the emodin- and chrysophanol-treated groups, total MCA and total UDCA in the emodin-treated groups were also significantly increased after 14-day treatment (Figure 4A). The concentration of total LCA was found significantly decreased in the bile and plasma but not changed in the liver after treating for 14 days (Figure 4B, Supplementary Figure S3). After treating for 28 days, accumulation of the total LCA in the liver was significantly increased in all the PMR- or anthraquinones-treated groups (Figure 4A), but not altered in the mouse bile and plasma (Figure 4B, Supplementary Figure S3). Furthermore, after treating for 14 days, the tested anthraquinones and PMR could not obviously affect the composition of amidated BAs in the mouse liver and bile. Unexpectedly, the percentage of amidated BAs in the mouse plasma was reduced by emodin treatment for 14 days, while chrysophanol significantly increased the percentage of amidated BAs in the mouse plasma. Moreover, the composition of amidated BAs in the mouse plasma was not obviously altered by either physcion or PMR extract (Supplementary Table S1). After 28-day treatment, except for the physcion, other two anthraquinones and PMR decreased the percentage of amidated BAs in the liver, while the percentage of amidated BAs in the bile and plasma was not changed after PMR or anthraquinones treatment (Supplementary Table S1).

### 3.4 PCA and PLS-DA Analysis

**FIGURE 5:**
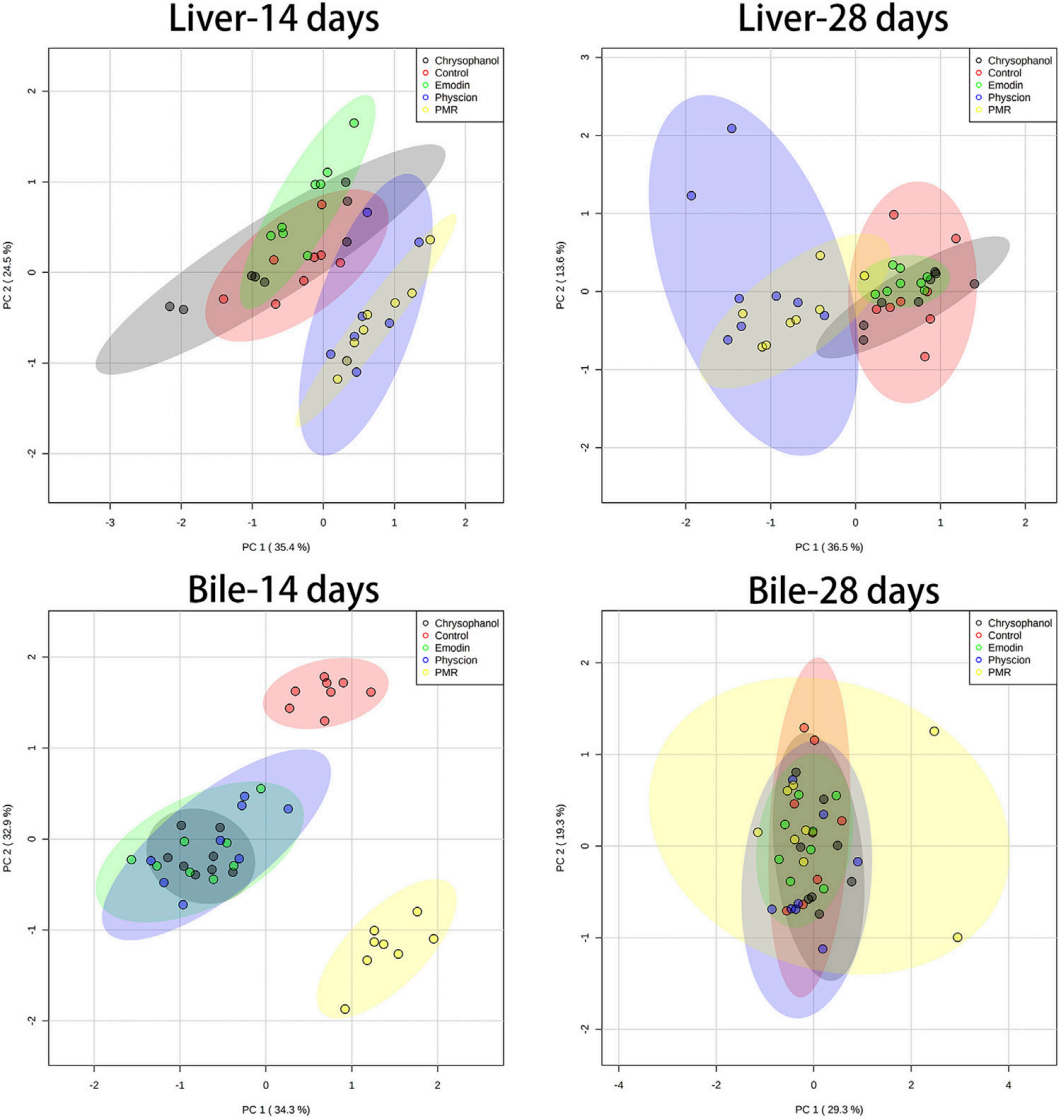
PCA results based on the quantitative analysis of the BAs in the mouse liver and bile from different groups of mice following 14- or 28-day treatment with anthraquinones or PMR extract.

**FIGURE 6:**
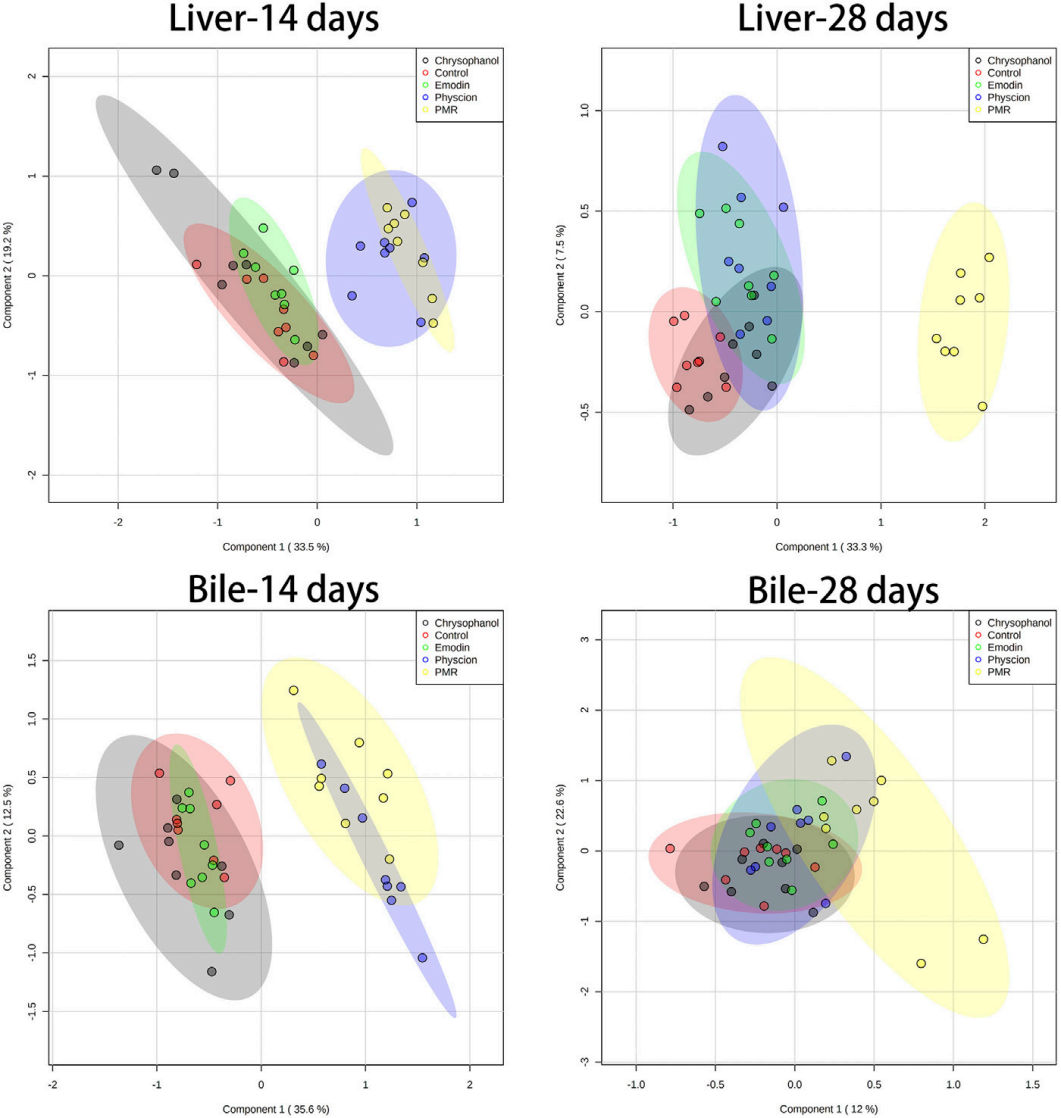
PLS-DA results based on the quantitative analysis of the BAs in the mouse liver and bile from different groups of mice following 14- or 28-day treatment with anthraquinones or PMR extract.

The datasets of the BA concentrations in different matrices from different treatment groups were used for PCA and PLS-DA analyses. The parameters used to assess the modelling quality included R2X, R2Y, and Q2Y and the values of R2 and Q2 were more than 0.5, indicating the successful establishment of the model. PCA was employed to reveal the BA disposition changes in different matrices. As shown in Figure 5, after treating the mice for 14 days, the PMR-treated groups showed a clear separation with the control group in the mouse bile and liver (Figure 5) on the score plot. In the meantime, all the treated groups showed a clear separation with the control group and PMR separated well with all the other anthraquinones groups after the 14-day administration in the bile (Figure 5). In the plasma, no separation was showed between the treated and control groups. With the administration duration prolonging to 28 days, the physcion treated groups showed a clear separation with the control group in the liver (Figure 5). The PLS-DA was adopted to evaluate and maximize the discrimination between the control and treated groups. As shown in Figure 6 and Supplementary Figure S4, a clear separation between the control and physcion/PMR treated groups was observed in the mouse liver, bile, and plasma after treating for 14 days. However, emodin and chrysophanol could not be separated with the control group on the PLS-DA score plot of bile in the liver, bile, and plasma after 14-day administration. With the administration duration prolonging to 28 days, the PMR group separated well with the control and the other three anthraquinones-treated groups in the liver. However, in the bile and plasma, no clear separation was observed among the control group and the treated groups after 28-day administration on the PCA or PLS-DA score plots (Figure 5, 6, Supplementary Figure S4).

### 3.5 Alteration of the Gene and Protein Levels of BA Transporters

**FIGURE 7:**
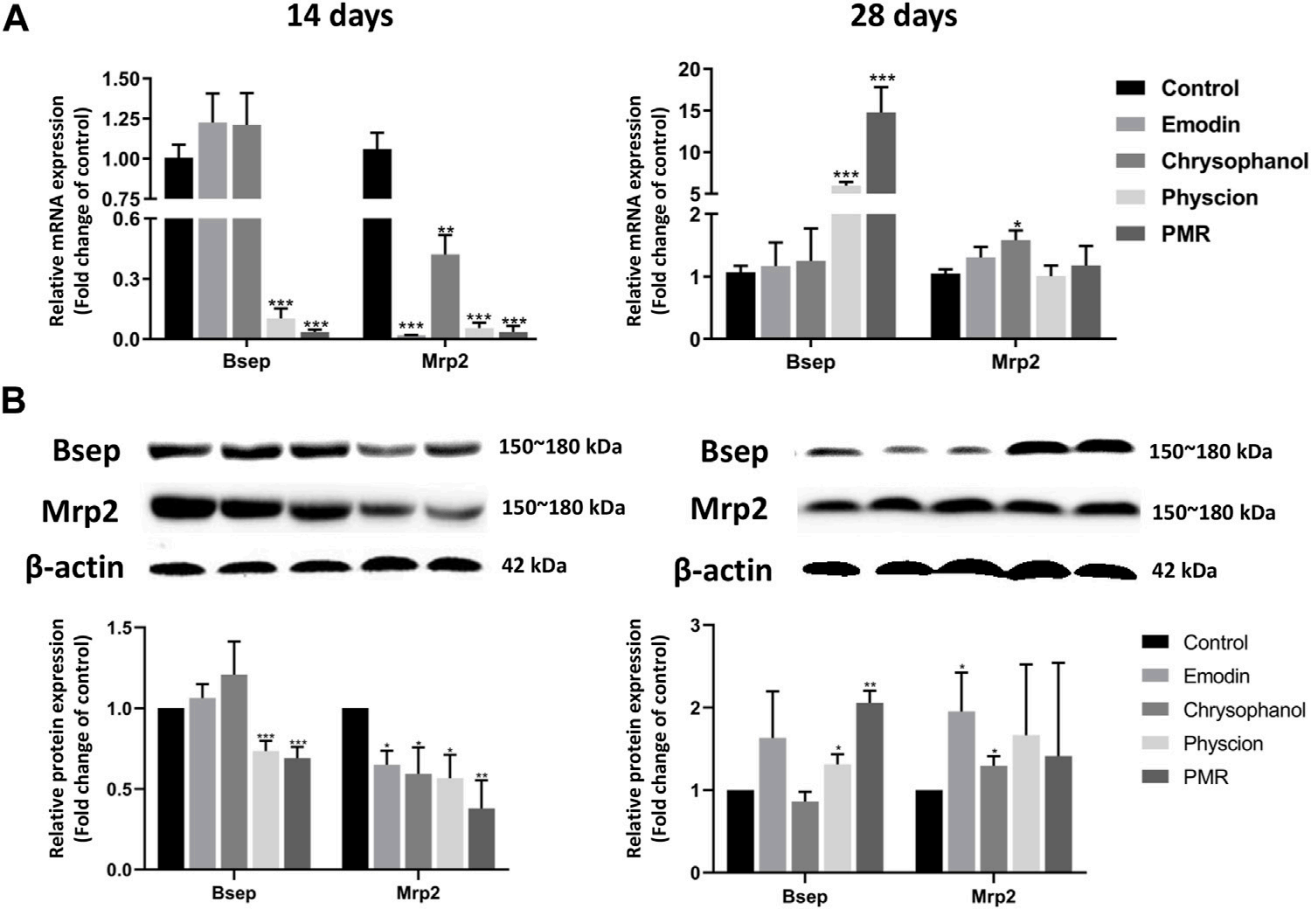
mRNA **(A)** and protein **(B)** expression levels of Bsep and Mrp2 in the mouse livers. Bar plots were represented as the mean ± SD (n = 8 for quantitative real-time PCR, n = 4 for western blotting using each two samples pooled). *p < 0.05, **p < 0.01, ***p < 0.001 compared with control group.

To explore the potential mechanisms of the influence of PMR on the homeostasis of BAs, the gene and protein expression of the key BA efflux transporters including Bsep and Mrp2 was investigated in our current study. As shown in Figure 7, after orally administering the mice with PMR extract or the anthraquinones for 14 days, the gene and protein expression of Mrp2 was significantly inhibited. In addition, the expression of Bsep was also significantly inhibited by the physcion and PMR extract. After treating the mice for 28 days, the gene and protein expression of Bsep was significantly increased in the physcion and PMR extract groups. Moreover, the expression of Mrp2 also significantly increased in the emodin- and chrysophanol-treated groups.

## 4 Discussion

BA homeostasis is strictly maintained by a series of uptake and efflux transporters as well as synthesis and metabolism enzymes (Rodrigues et al., 2014). Experimental toxicology studies have identified the disturbed metabolism of BAs as a common and early event of drug-induced liver injury by diverse hepatotoxins (Yamazaki et al., 2013). As indicated by the previous studies, PMR induced liver injury was closely associated with BA homeostasis disruption. In the current work, we explored the hepatotoxic effect of three major PMR-derived anthraquinones including emodin, chrysophanol, and physcion as well as the PMR ethanol extract based on BA disposition and complemented with serum biochemical indicators and histopathological examination in the mice. All these three anthraquinones and the PMR extract were found to induce liver injury as evidenced by the histopathological examination and the hepatotoxic biomarker levels in the serum. Additionally, it was found that all the anthraquinones of interest and PMR extract could alter the BA homeostasis.

To uncover any potential toxicity of the PMR extract or the anthraquinones, the doses were set at high levels and the dose of PMR was around 25-fold the highest recommended human dose obtained from the 2015 edition of the Chinese Pharmacopoeia (the dose of human was converted to mice based on the body surface area conversion (US Food and Drug Administration, 2005). According to the previous reports, there is an interaction between the co-occurring components in PMR. Xing et al. (2019) have evidenced that the C_max_ of emodin in the rat plasma could be increased by 3–7 times in the presence of TSG. In our current study, the dose of PMR was selected as 30 g/kg, which was equivalent to 14.1 mg/kg emodin. To mimic the maximum exposure of emodin after administration with PMR extract, the dose of emodin was selected as 100 mg/kg (14.1 mg/kg × 7 = 98.7 mg/kg). In order to compare the impact of these anthraquinones parallelly, the dose of other two anthraquinones, chrysophanol and physcion, was also selected as 100 mg/kg.

Currently, the serum biochemical parameters such as ALT, AST, and ALP are used as conventional biomarkers for the clinical diagnosis (Park et al., 2001; Jung et al., 2011; Dong et al., 2014). In our study, the AST levels were significantly increased after treating for 28 days with the PMR extract or the pure anthraquinones, which correlated well with the histopathological findings. However, neither anthraquinones nor the PMR extract altered the biomarkers tested in our current study including ALT, AST, TG, and γ-GGT after 14-day treatment, while the distinct results were observed that physcion and PMR caused liver damage after 14-day treatment evidenced by the liver histopathological changes. Such discrepancy may be resulted from the limited sensitivity and specificity of ALT and AST in the serum for early detection of liver injury (McGill, 2016). Although, ALT was suggested to be a more sensitive biomarker for liver injury in clinical practice (Senior, 2009), the significant elevation of AST rather than ALT was observed in all the treatment groups in our current study and the previous report in which the rats were treated with the PMR extract (equivalence to 20 g/kg crude material) for 21 days (Ma et al., 2015). Such discrepancy could be due to the species difference between the rodents and humans or AST was suggested to be a more sensitive biomarker for PMR-induced liver injury. McGill reviewed the mechanisms and interpretation of the plasma levels of ALT and AST elevation in liver injury and stated that ALT and AST have poor prognostic utility in acute liver injury and liver failure (McGill, 2016). In addition, physcion was reported to be cytotoxic to red blood cells by triggering hemolysis and programmed cell death (Akiel et al., 2021). As a result, unpredictable response to the different degree of hemolysis might also affect the accurate measures of such biochemical testing (Lippi et al., 2006).

In preclinical studies, the change of BAs was also used as an important physiological indicator of liver damage (Casini et al., 1997) and several reports have also recommended the GDCA in the bile, hyodeoxycholic acid (HDCA) in the plasma, or TMCA in the urine as potential biomarkers for PMR induced liver injury in the SD rats (Dong et al., 2015; Zhao et al., 2017). In the current study, the total BAs in the liver were significantly increased accompanied with the significant decrease in the bile after treating with physcion and PMR for 14 days. These results correlated well with the histopathological results that the liver obtained from these two groups showed typical inflammatory cell infiltration. After 28-day treatment, it was noted that the accumulation of the total BAs in the liver was decreased in the physcion treatment group, which may be related to the adaptive protective mechanisms (Zollner et al., 2006). However, such a reduction of total BAs in the liver did not prevent the occurrence of liver damage evidenced by the histopathological findings. A plausible explanation for such hepatic toxicity was that the accumulation of physcion in the liver resulted from long-term treatment leading to hepatocellular damage. It was found that physcion could induce significant cytotoxic effects against the human liver cell line L02, HepG2 cells, rat primary hepatocytes, and sandwich-cultured rat hepatocytes in a dose dependent manner (Xun et al., 2019).

The toxicity of the BAs is related to their structures and the hydrophobic BAs is more toxic than the hydrophilic BAs (LCA ≥ DCA ≥ CDCA >> CA) (Trottier et al., 2006; Schadt et al., 2016). The accumulation of hydrophobic BAs in the liver can result in mitochondrial dysfunction by generating reactive oxygen species (ROS), which in turn causes liver injury (Casini et al., 1997; Perez and Briz, 2009). In our study, it was worth noting that after 14-day or 28-day administration with anthraquinones or PMR extract, the hydrophobic BAs (DCA, CDCA, and CA) in the liver were significantly accumulated. Moreover, the significant accumulation of the total LCA was also observed in the liver after treating the mice for 28 days. BA conjugation with glycine or taurine could increase the solubility and decrease the toxicity of the corresponding BAs (Trottier et al., 2006). The significantly decreased proportion of the conjugated BAs in the liver, at least in part, indicated that toxic BA detoxification could be altered by PMR and typical anthraquinones. Taken together, the accumulation of these hydrophobic BAs and inhibition of BA conjugation were considered to contribute to the liver toxicity induced by PMR and anthraquinones of interest. This might also be a possible explanation for the discrepancy between the attenuation of liver total BA accumulation in the physcion treatment group and the liver damage evidenced by histological examination after treating for 28 days.

In human, BAs undergo sulphation to reduce their cytotoxicity (Agellon and Torchia, 2000; Zwicker and Agellon, 2013). Sulfotransferase-2A1(SULT2A1) is the enzyme catalyzing the BAs into BA-sulfates, which are more hydrophilic and readily excreted into feces and urine (Alnouti, 2009). According to previous study, the activity and expression of SULT2A1 could be significantly inhibited by emodin or rhein in the HepG2 cells (Maiti et al., 2014). The inhibition of SULT2A1 might directly contribute to the decrease of liver BA-sulfates level in our current study. Moreover, hepatic transporters also play critical roles in maintaining the BA homeostasis (Agellon and Torchia, 2000). BAs were synthesized from cholesterol in the hepatocytes and secreted across the canalicular membrane in an ATP-dependent fashion *via* BSEP/Bsep and MRP2/Mrp2 (Akita et al., 2001). Since canalicular secretion is considered as the rate-limiting step of the vectorial export of toxic BAs from hepatocytes into bile, impaired BA biliary transport may directly result in hepatic BA accumulation. In addition, basolateral BA efflux transporters, Mrp3 and Mrp4, are also involved in the BA homeostasis and the inhibition of both canalicular and basolateral BA efflux transporters may worsen liver injury (Corsini and Bortolini, 2013; Köck et al., 2014). In our current study, the significant decrease of gene and protein expression of Bsep in the physcion and PMR groups was supposed to be the major factor of the hepatic total BA accumulation after treating for 14 days. After 28 days of treatment, hepatic total BA accumulation was attenuated due to the elevated mRNA and protein expression of Bsep in the physcion group. However, the increased accumulation of hepatic total BAs was contradictory with the significant elevation of Bsep after treatment with PMR extract for 28 days. Such discrepancy could be explained by the concurrent inhibition of the basolateral BA efflux induced by these three anthraquinones in PMR as demonstrated in our previous study (Kang et al., 2017). Unlike Bsep, the inhibition of Mrp2 may not induce the accumulation of hepatic BAs (Iyanagi et al., 1998), which was consistent with our results that the altered protein expression of Mrp2 showed no impact on the hepatic total BA levels in the emodin and chrysophanol treated groups. Furthermore, the inconsistency of the changed expression of Bsep and Mrp2 with the individual hepatic BAs could be due to their distinct affinity to such efflux transporters (Noe et al., 2001). Compared with the control group, the total BAs in the bile were decreased by around 86% and 85%, while the total hepatic BA level was increased by 3.1- and 1.7-fold in the physcion- and PMR-treated groups, respectively. However, in agreement with the previous reports (Wang et al., 2015; Zhao et al., 2017), the expression of BA efflux transporters, Bsep and Mrp2, was increased with the administration prolonging to 28 days, which was in accordance with the disposition of BAs that the percentage of amidated BAs in the liver, the major substrates of Bsep, was significantly decreased. Since the individual BAs such as CDCA, DCA, CA, and LCA are the agonists of Farnesoid X receptor (FXR/Fxr) with CDCA as the most potent one (Stofan and Guo, 2020), the accumulated BAs could activate FXR/Fxr and therefore inhibit the synthesis of BAs and induce the expression of Bsep to attenuate the overload of hepatic BAs (Schaap et al., 2014). Moreover, CA and UDCA were reported to induce the expression Mrp2 with a Fxr-independent manner in the mice (Zollner et al., 2003). Therefore, the regulation of the expression of the two transporters in our current study could be explained as the adaptive response for limiting the hepatic BA overload (Zollner et al., 2006). Additionally, the decrease of part of the BAs in the plasma in our study may result from the decreased reabsorption of BAs due to the inhibition of the excretion of BAs into the bile as well as the inhibition of the bile acid efflux transporters Mrp3 and Mrp4. Also, the plasma BA concentrations could be affected by the hepatocellular uptake from the blood evidenced by the downregulated expression of major hepatic BA uptake transporter, sodium taurocholate cotransporting polypeptide (Ntcp) in our previous study (Kang et al., 2017). In addition, renal elimination, hepatic synthesis and metabolism, and intestinal metabolism and elimination from feces of BAs have also been recognized as another vital factor for the BA levels in the plasma. Therefore, the complex regulation processes might be a plausible reason for the relatively minor alteration of BAs in the plasma.

Taken together, the present study comprehensively elucidated the role of PMR or its major anthraquinones in BA disposition. It was found that PMR or physcion could induce hepatic BA accumulation after treating for 14 days, which were potentially associated with the inhibition of the function and expression of hepatic BA canalicular efflux transporters, Bsep and Mrp2, as well as the basolateral efflux transporters, Mrp3 and Mrp4. Such cholestasis could be attenuated *via* regulating the expression of BA transporters with a prolonging administration duration up to 28 days. This study, for the first time, investigated the hepatotoxicity of the major anthraquinones from PMR based on BA homeostasis and found that all the tested anthraquinones of interest could change the disposition of the BAs to a different extent in mice. However, in addition to anthraquinones, there are many other components such as stilbene, tannins, dianthrones, and flavonoids in the PMR and the stilbene was reported to increase the systemic exposure of emodin *via* increasing its absorption and inhibiting its metabolism (Li et al., 2020b). That could also be a possible explanation that the hepatoxicity of other traditional Chinese medicine containing anthraquinones was less reported than PMR. It is also worth noting that dianthrones derivatives, such as (*Cis*)-emodin-emodin dianthrones and (*Tran*s)-emodin-emodin, have recently been reported to be associated with PMR hepatotoxicity (Yang et al., 2018a; Li et al., 2020). Furthermore, the mutual transformation between the dianthrones derivatives and anthraquinones results in more complicated potential toxic source analysis for PMR induced liver injury (Yang et al., 2018a). Therefore, the influence of the interaction between anthraquinones and other components from PMR on the BA steady-state should be taken into further consideration for the quality control and safe use of PMR in practice. In addition, due to the species difference between the mice and human, the translation of the current results to humans warrants further investigation.

## 5 Conclusion

Both the PMR extract and three anthraquinones of interest could alter the disposition of either the total or individual BAs in the mouse bile, liver, and plasma. Physcion and PMR extract treatment elevated the hepatic total BAs and decreased that in the bile, while it could be attenuated with prolonged administration duration. Hepatic accumulation and biliary excretion of most of the individual BAs were affected by the PMR extract and three anthraquinones. Such alteration was potentially related with the inhibition on the function and expression of dominant BA efflux transporters.

## Data Availability

The original contributions presented in the study are included in the article/Supplementary Material, Further inquiries can be directed to the corresponding authors.

## Glossary

ACN: Acetonitrile
ALT: Alanine aminotransferase
AST: Aspartate aminotransferase
BAs: Bile acids
BCA: Bicinchoninic acid
Bsep: Bile salt export pump
CA: cholic acid
CDCA: Chenodeoxycholic acid
CA-S: Cholic acid-sulfate
CMC-Na: Carboxymethylcellulose sodium
d4-GCDCA: Deuterium-labeled glycochenodeoxycholic acid
DCA: Deoxycholicacid
Fxr: Farnesoid X receptor
GCA: Glyco-cholic acid
GMCA: Glyco-β-muricholic acid
H&E: Hematoxylin and eosin
HDCA: Hyodeoxycholic acid
LCA: Lithocholic acid
LC-MS/MS: Liquid chromatography with tandem mass spectrometry
MeOH: Methanol
Mrp2: Multidrug resistance-associated protein 2
PLS-DA: Partial Least Squares Discriminate Analysis
PMR: Polygoni Multiflori Radix
ROS: Reactive oxygen species
Ntcp: Sodium taurocholate cotransporting polypeptide
SULT2A1: Sulfotransferase-2A1
TCA: Tauro-cholic acid
TCA-S: Tauro-cholic acid-sulfate
TCDCA: Tauro-chenodeoxycholic acid
TCDCA-S: Tauro-chenodeoxycholic acid-sulfate
TDCA: Tauro-deoxycholic acid
TG: Triglyceride
TLCA: Tauro-lithocholic acid
TLCA-S: Tauro-lithocholic acid-sulfate
TMCA: Tauro-β-muricholic acid
TUDCA: Tauro-ursodeoxycholic acid
TSG: 2,3,4′,5-tetrahydroxystilbene 2-*O*-*β*-D-glucoside
UDCA: Ursodeoxycholic acid
UDCA-S: Ursodeoxycholic acid-sulfate
MCA: β-muricholic acid
MCA-S: β-Muricholic acid-sulfate
γ-GGT: γ-glutamine transferase
