# Supplementary material for: Hepatotoxicity of the Major Anthraquinones Derived From Polygoni Multiflori Radix Based on Bile Acid Homeostasis
Source: Front Pharmacol. 2022 May 18;13:878817. doi: 10.3389/fphar.2022.878817 (PMC9157432; doi:10.3389/fphar.2022.878817)
Supplement: Supplementary file 1 [file DataSheet1.docx]

Supplementary Material


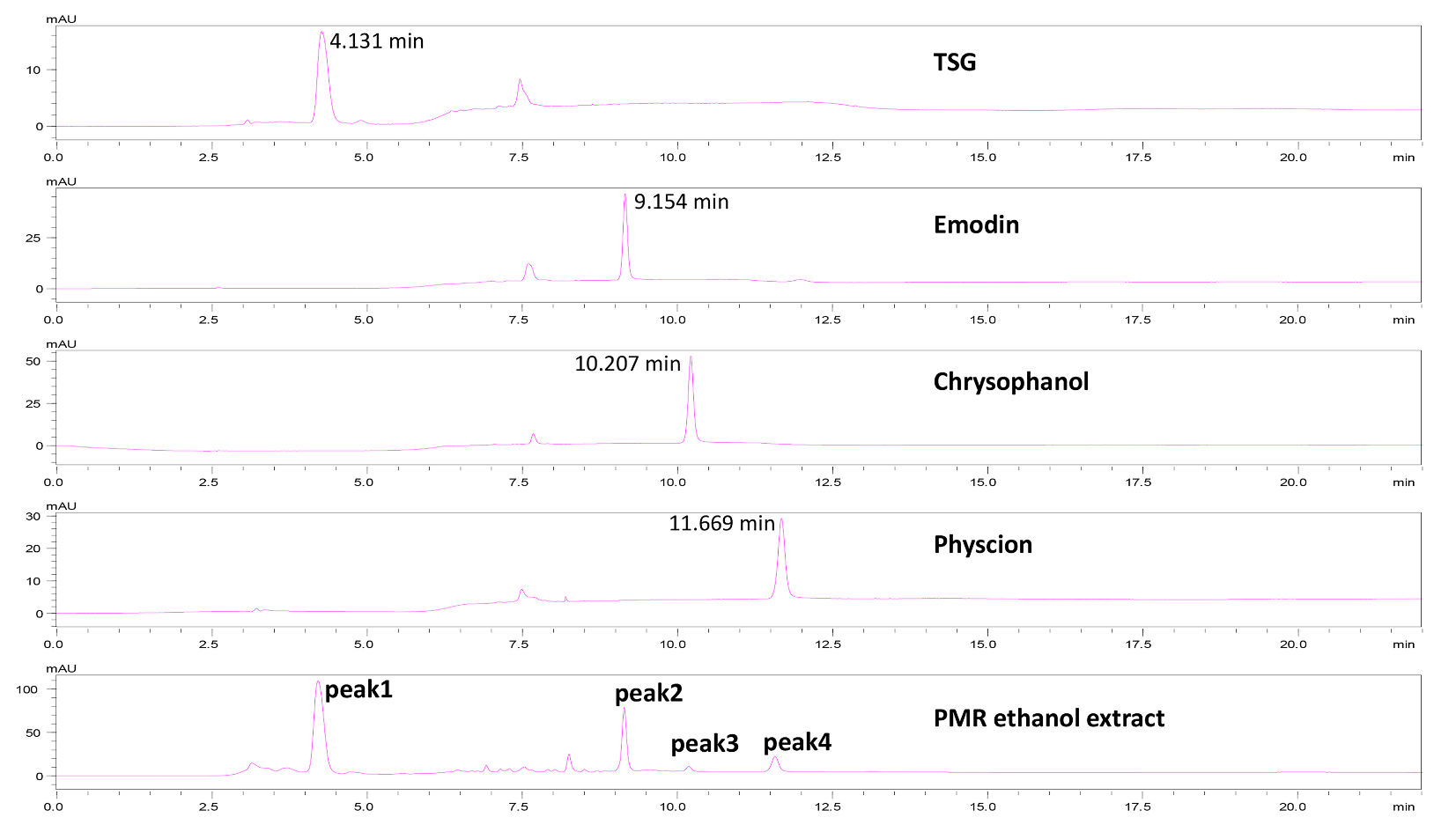


**Figure S1**. Representative chromatograms of typical anthraquinones and PMR ethanol extract. Peak assignments: 1, 2,3,4’,5-tetrahydroxystilbene-2-*O*-*β*-D-glucoside (TSG); 2, emodin; 3, chrysophanol and 4, physcion


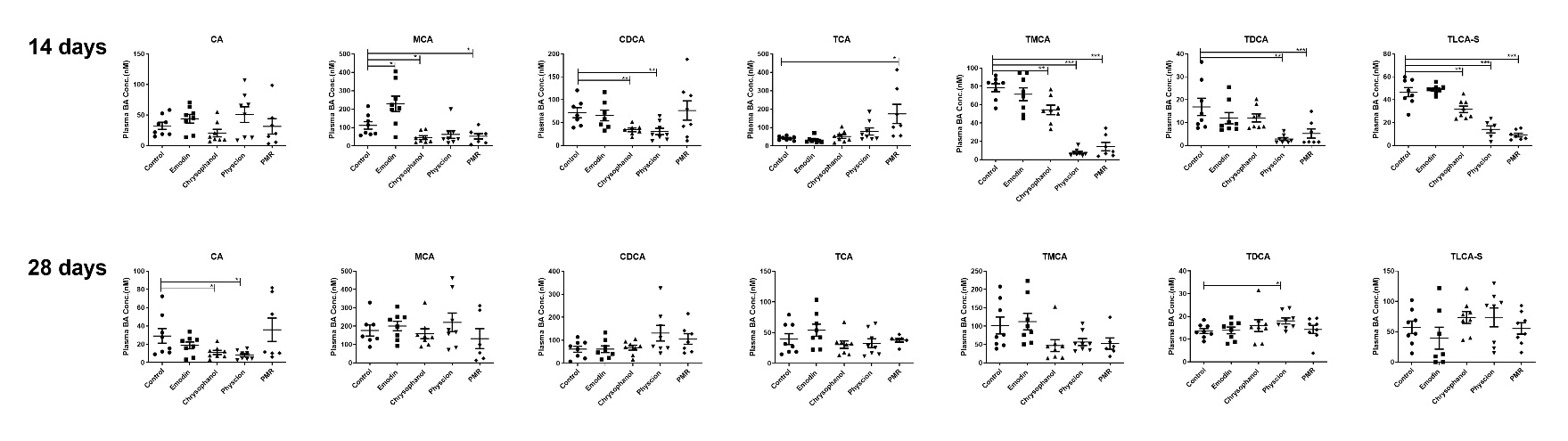


**Figure S2**. Concentration of the individual BAs in mouse plasma after the anthraquinones or PMR consumption for 14 or 28 days. The data are expressed as the mean ± SD (n =6-8). **p* < 0.05, ***p* < 0.01, ****p* < 0.001 compared with control group.


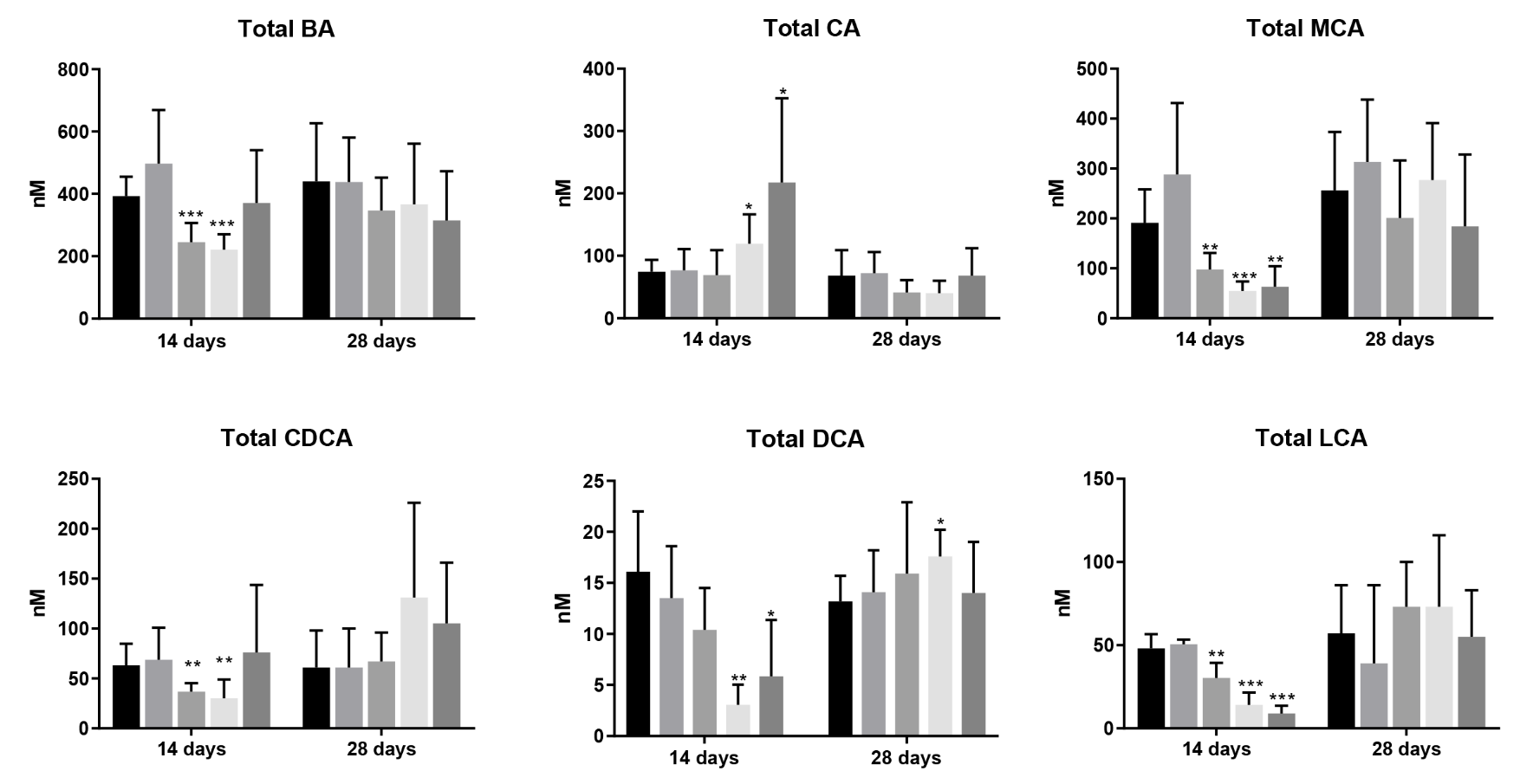


**Figure S3**. Comparison of the total BA, total CA, total MCA, total CDCA, total UDCA, total DCA and total LCA in the mouse plasma between different groups of mice following 14- or 28-day treatment with anthraquinones or PMR extract. The data are expressed as the mean ± SD (n =6-8). **p* < 0.05, ***p* < 0.01, ****p* < 0.001 compared with control group.


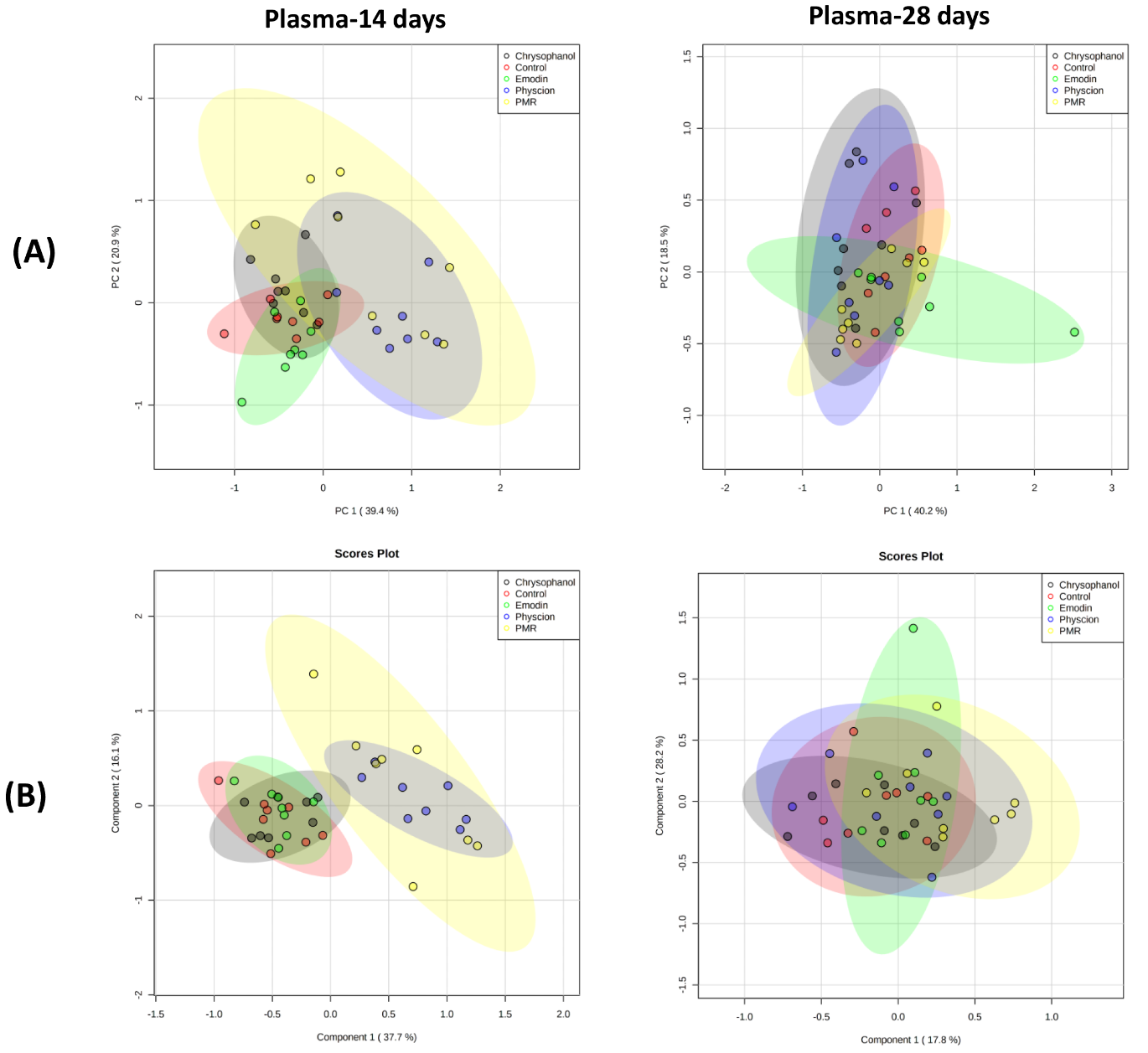


**Figure S4**. PCA (A) and PLS-DA (B) results based on the quantitative analysis of the BAs in the plasma from different groups of mice following 14- or 28-day treatment with anthraquinones or PMR extract.

**Table S1.** Percentage of the amidated BAs in the liver, bile and plasma from different groups of mice following 14- or 28-day treatment after treating with anthraquinones or PMR extract (n=6-8)

| Group | | Control | | Emodin | | Chrysophanol | | Physcion | | PMR | |
| --- | --- | --- | --- | --- | --- | --- | --- | --- | --- | --- | --- |
|  |  | Mean | SD | Mean | SD | Mean | SD | Mean | SD | Mean | SD |
| Liver | 14 days | 93.502 | 3.797 | 91.572 | 2.396 | 93.129 | 1.920 | 93.215 | 5.905 | 94.322 | 2.629 |
|  | 28 days | 90.536 | 1.986 | 85.132^**^ | 3.783 | 85.682^**^ | 3.803 | 87.523 | 5.838 | 88.031^**^ | 0.111 |
| Bile | 14 days | 99.580 | 0.242 | 99.445 | 0.207 | 99.213 | 3.165 | 99.424 | 0.170 | 99.213 | 2.355 |
|  | 28 days | 99.598 | 0.167 | 99.659 | 0.108 | 99.689 | 0.162 | 99.727 | 0.097 | 99.681 | 0.112 |
| Plasma | 14 days | 48.479 | 5.356 | 31.516^*^ | 9.035 | 62.362^*^ | 23.822 | 43.615 | 23.645 | 54.596 | 30.655 |
|  | 28 days | 41.576 | 15.292 | 38.538 | 9.412 | 37.206 | 15.496 | 31.837 | 16.718 | 40.297 | 13.412 |

**p* < 0.05, ***p* < 0.01, ****p* < 0.001 compared with control group.
